# Increased Epicardial Adipose Tissue and Heart Characteristics Are Correlated with BMI and Predict Silent Myocardial Infarction in Sudden Cardiac Death Subjects: An Autopsy Study

**DOI:** 10.3390/diagnostics13132157

**Published:** 2023-06-24

**Authors:** Timur Hogea, Nagy Noemi, Bogdan Andrei Suciu, Klara Brinzaniuc, Laura Chinezu, Emil Marian Arbănași, Réka Kaller, Cosmin Carașca, Eliza Mihaela Arbănași, Vlad Vunvulea, Ioana Hălmaciu, Adrian Vasile Mureșan, Eliza Russu, Claudiu Constantin Ciucanu, Casandra Maria Radu, Corina Carmen Radu

**Affiliations:** 1 Department of Forensic Medicine, George Emil Palade University of Medicine, Pharmacy, Science, and Technology of Targu Mures, 540139 Targu Mures, Romania; timur.hogea@umfst.ro (T.H.); carmen.radu@umfst.ro (C.C.R.); 2 Institute of Forensic Medicine, 540141 Targu Mures, Romania; ioana.halmaciu@umfst.ro; 3 Doctoral School of Medicine and Pharmacy, George Emil Palade University of Medicine, Pharmacy, Science, and Technology of Targu Mures, 540142 Targu Mures, Romania; emilarbanasi1@gmail.com (E.M.A.); reka.kaller@umfst.ro (R.K.); 4 Department of Anatomy, George Emil Palade University of Medicine, Pharmacy, Science, and Technology of Targu Mures, 540139 Targu Mures, Romania; bogdan.suciu@umfst.ro (B.A.S.); klara.brinzaniuc@umfst.ro (K.B.);; 5 Department of Histology, George Emil Palade University of Medicine, Pharmacy, Science, and Technology of Targu Mures, 540139 Targu Mures, Romania; laura.chinezu@umfst.ro; 6 Clinic of Vascular Surgery, Mures County Emergency Hospital, 540136 Targu Mures, Romania; eliza.russu@umfst.ro (E.R.); adrian.muresan@umfst.ro (A.V.M.); 7 Department of Vascular Surgery, George Emil Palade University of Medicine, Pharmacy, Science, and Technology of Targu Mures, 540139 Targu Mures, Romania; 8 Faculty of Pharmacy, George Emil Palade University of Medicine, Pharmacy, Science, and Technology of Targu Mures, 540139 Targu Mures, Romania; arbanasi.eliza@gmail.com; 9 Department of Radiology, Mures County Emergency Hospital, 540136 Targu Mures, Romania; 10 Doctoral School of Biological and Biomedical Sciences, University of Oradea, 1 University Street, 410087 Oradea, Romania

**Keywords:** silent myocardial infarction, coronary artery disease, sudden cardiac death, forensics, risk factors, epicardial adipose tissue

## Abstract

Background: Sudden cardiac death (SCD) is a significant global public health issue and the leading cause of death worldwide. Its etiopathogenesis is complex and multilayered, involving dynamic factors interacting with a preexistent cardiovascular pathology, frequently unknown, and resulting in cardiac rhythm disorders and cardiac arrest; Methods: This study conducted a retrospective descriptive analysis over a one-year period, identifying 321 autopsy cases of sudden deaths from the Institute of Legal Medicine in Mures County, Romania, in 2019. From the 321 sudden death cases, 189 autopsy reports were selected for analysis based on inclusion and exclusion; Results: The autopsies had a mean age of 61.16 years and included 140 males and 49 females. No significant differences were found between the silent myocardial infarction (SMI) and no-SMI groups regarding demographic data. The SMI group exhibited higher thickness of LV (left ventricle), IV (interventricular septum), EAT LCx (epicardial adipose tissue at left circumflex artery), EAT LAD (epicardial adipose tissue at left anterior descending artery), heart weight, and BMI (body mass index). The left coronary artery showed a higher incidence of type V plaques, while the right coronary artery showed higher incidences of type V and type VI plaque. The SMI group also exhibited a higher incidence of moderate and severe valvular atherosclerosis, severe left ventricle dilatation, and a lower incidence of mild left ventricle dilatation. In addition, the SMI group showed a higher presence of contraction band necrosis on histological examination. Multivariate analysis revealed that type V and type VI plaques for the right and left coronary arteries, moderate and severe valvular atherosclerosis, severe left ventricle dilatation, heart weight, EAT LCx, EAT LAD, LV thickness, IV thickness, BMI, and the presence of contraction band necrosis are all independent predictors of SMI; Conclusions: The findings suggest that SCD is a complex condition, and its etiopathogenesis involves dynamic factors interacting with pre-existing cardiovascular pathology. The risk factors of SCD are similar to those of ischemic heart disease. The findings of this study could guide clinicians in identifying patients at risk of SCD and implementing preventive measures.

## 1. Introduction

Sudden cardiac death (SCD) represents a natural death, predominantly with a known or unknown cardiovascular etiology, signaled by an abrupt loss of consciousness, with death occurring in under one hour from the onset of the symptoms if no proper resuscitation maneuvers are immediately deployed [1]. If a healthy individual dies suddenly and unexpectedly, in a public place or at home, with no witnesses, suspicions arise in the justice system that it was a violent death. The term SCD reflects a sudden stop of cardiac activity followed, after several minutes, by cerebral death [2]. According to the WHO, it is still regarded as a global public health issue with few to no viable solutions available, being ranked as the leading cause of death worldwide [3]. The etiopathogenesis of SCD has a complex and multilayered substrate in which dynamic factors interact with a preexistent cardiovascular pathology, frequently unknown, leading to the development of cardiac rhythm disorders (most often, ventricular tachycardia that evolves into ventricular fibrillation) and cardiac arrest. SCD risk factors are identical to those of ischemic heart disease: old age, male gender, family history of ischemic heart disease, obesity, high LDL cholesterol, diabetes, hypertension, abuse of alcohol, coffee, and/or caffeinated energy drinks, smoking, sudden high-intensity physical activity, substance abuse, inherited genetic abnormalities (long QT syndrome, Brugada syndrome, obstructive hypertrophic cardiomyopathy) [4,5,6,7,8,9].

The prevalence of SCD has two peaks: the first in the population under 35 years (inherited genetic rhythm disorders or cardiomyopathies, abuse of arrhythmogenic substances and/or caffeinated energy drinks, juvenile smoking, and high BMI with high LDL cholesterol), and the second for the 45–75 year interval (most often coronary atherosclerosis, cardiomyopathies, myocardosclerosis, silent myocardial infarction with scarring). In the second peak, the forensic autopsy frequently identifies multiple cardiovascular pathologies, making it difficult to establish which one is responsible for the SCD. In the young population, the cause of SCD can remain unknown even after the forensic autopsy because cardiac rhythm disorders do not leave a macroscopic trace and are simply presumed in a so-called „white autopsy”. Studies have shown that an alarming 80% of cases above 35 years of age present high degrees of coronary atherosclerosis [10,11]. In the last decades, cardiovascular researchers have struggled to comprehend the complex mechanisms of SCD, primarily identifying risk factors for the patients who suffered a heart attack and lived, stratifying them, and deploying medical treatment (beta blockers, statins, thrombolytic and antithrombotic agents, angiotensin-converting enzyme inhibitors), with a major benefit from implantable cardioverter-defibrillators (ICD) overall reducing to some degree the mortality of cardiac causes [12,13,14,15].

Two major groups with acute myocardial infarction (MI) have not benefited from these new protocols: those who presented SCD as the first and only manifestation of an MI and those who had been asymptomatic or presented atypical symptoms and disregarded the acute event [16]. For patients with silent myocardial infarction (SMI), the myocardial scar creates an arrhythmogenic substrate prone to fatal arrhythmias [17], thus exponentially increasing the risk for an SCD [17,18,19]. We found a scarcity of data regarding the prevalence and role of an SMI in SCD cases.

The present study aims to expand our previous research by determining the prevalence and characteristics of SMI among all SCD cases that were autopsied over one year at the Institute of Forensic Medicine in Targu Mures, Romania, and analyzing the predictor role of epicardial adipose tissue, hearth characteristics, coronary plaque morphology, body mass index (BMI), and SMI risk in SCD patients.

## 2. Materials and Methods

### 2.1. Study Design

We conducted a retrospective descriptive analysis over a one-year period, identifying 321 autopsy cases of sudden deaths from the Institute of Legal Medicine in Mures County, Romania, in 2019.

### 2.2. Inclusion Criteria

From the 321 identified sudden death cases we only selected 189 autopsy reports with the cause of death related to cardiovascular pathologies (Caucasians; 74.07% male, 25.93% female; 33–87 years old) who died suddenly in apparent good health (no prior known cardiovascular pathologies), in under 24 h from the onset of any symptoms (if they were present), with or without witnesses, no matter where the death occurred or if ICU measures were applied.

### 2.3. Exclusion Criteria

We excluded patients that had previously been identified with cardiovascular diseases, with or without medical treatment, sudden deaths from other non-cardiac causes, and those discovered in an advanced condition of autolysis or putrefaction from the outset. Autopsies in which toxicology reports revealed the presence of alcohol, narcotics, illicit substances, poisons, or other chemical compounds at deadly dosages were also excluded. No toxicology exams were performed.

### 2.4. Studied Parameters

In this retrospective study, we considered age, gender, BMI, urban/rural area of the cases, and the place of death. Macroscopic cardiovascular pathological findings were evaluated during the autopsy by two forensic doctors.

On all autopsies performed according to the European guidelines of SCD [20], our standard protocol involved: weighing of the heart with an abnormal weight > 0.5% of BMI or above 500 g, thickness measurement of the ventricular walls (lateral walls of the left/right ventricles and interventricular wall), with an abnormal thickness ≥ 15 mm or <2 mm, thickness measurement of the epicardial adipose tissue (EAT) corresponding to the two left coronary branches.

The severity of left ventricle (LV) dilatation was determined using the American Society of Echocardiography criteria based on the LV internal diameter [21]: normal (42 to 59 mm for males; 39 to 53 mm for females), mildly dilated (60 to 63 mm for males; 54 to 57 mm for females), moderately dilated (64 to 68 mm for males; 58 to 61 mm for females), and severely dilated (>68 mm). The highest value of the LV transverse internal diameter was noted. Coronary and aortic atherosclerosis was subjectively evaluated by two forensic doctors and then determined by the histopathological examination that followed the autopsy.

The final extent of atherosclerosis was noted according to the histological grading of the American Heart Association Committee on Vascular Lesions of the Council on Arteriosclerosis [22], as follows: initial lesion (type I), progression-prone and resistant lesion (type II), pre-atheroma (intermediate lesion) (type III), atheroma (type IV), fibroatheroma (type Va), calcific lesion (type Vb), fibrotic lesion (type Vc), and lesion with the presence of a surface defect, hematoma, hemorrhage, or thrombotic deposit (type VI). Valvular atheromas were macroscopically graded using the following scale: absent (no deposits), mild (<3 mm spot-like deposits), moderate (>3 mm deposits ±calcifications), severe (extensive calcifications with mobility loss) [23].

**Figure 1:**
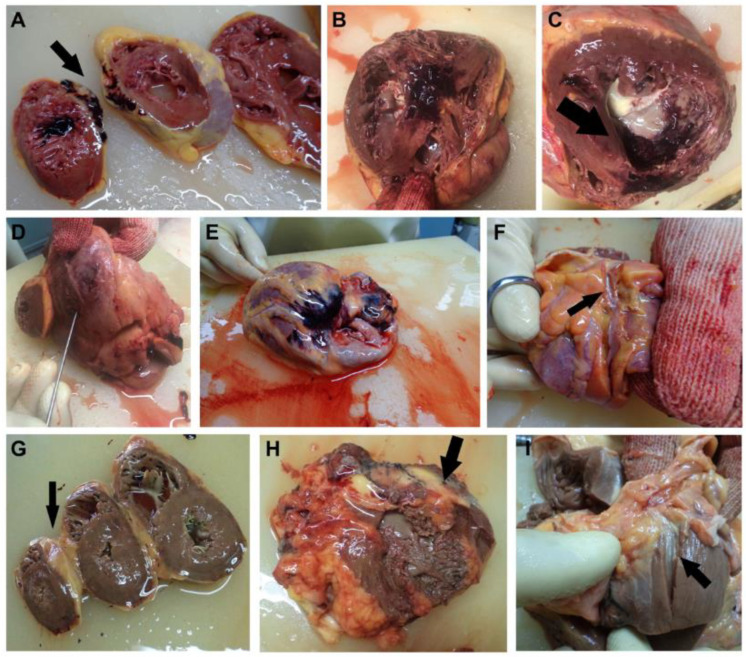
(**A**) Acute MI superimposed on a posterior septal silent myocardial infarction. (**B**) Acute MI overposed on a septal silent myocardial infarction with hypertrophic cardiomyopathy and myocardial sclerosis. (**C**) Extensive acute MI overposed on a posterior septal silent myocardial infarction with hypertrophic cardiomyopathy and myocardosclerosis. (**D**) Acute MI of the postero-lateral wall of the LV with myocardial rupture, hemopericardium, and cardiac tamponade (1100 mL blood in the pericardial sac). (**E**) Extensive acute MI overposed on an SMI in the anterolateral wall of the RV. (**F**) Hypoplasia of the circumflex artery (0.2 cm diameter at emergence, with sinuous trajectory). (**G**) Cardiac lipomatosis. The postero-lateral wall of the RV is replaced by adipose tissue. (**H**) Fibrinous pericarditis. The tight adhesion of the anterolateral wall of the RV to the pericardium resulted in an RV wall rupture during the autopsy (without hemorrhagic infiltration). (**I**) The Cx branch with hypoplasia and bridging (0.3 cm in the myocardium).

Myocardosclerosis, fibrosis, myocardial infarction scars from SMI, acute ischemia, acute myocardial infarction on the SMI scar or different location with or without wall rupture, hypoplastic coronary artery disease, cardiac lipomatosis, fibrinous pericarditis, coronary thrombosis, bridging, or hemorrhage was macroscopically determined, topographically described, and later confirmed by our pathologist (Figure 1). Histopathological examinations were performed from cardiovascular tissue fragments fixed in 10% formaldehyde for at least 24 h, with paraffin sections being stained with standard hematoxylin/eosin.

### 2.5. Statistical Analysis

From the collected data statistical analyses were performed using SPSS for Mac OS version 28.0.1.0 (SPSS, Inc., Chicago, IL, USA). Continuous variables are presented as mean ± standard deviation and categorical variables as the observed number of included cases. Chi-squared tests were used to assess the associations of the ratios with category factors, while Student’s t or Mann–Whitney tests were used to assess differences in continuous variables. To analyze the predictive power and to establish the cut-off values of BMI, heart weight, EAT LCx (epicardial adipose tissue at left circumflex artery), EAT LAD (epicardial adipose tissue at left anterior descending artery), the receiver operating characteristic (ROC) curve analysis was utilized. The ROC curve analysis was used to determine the appropriate BMI, heart weight, EAT LCx, and EAT LAD cut-off values based on the Youden index (Youden Index = Sensitivity + Specificity − 1, ranging from 0 to 1). To identify independent predictors of sudden cardiac death, a multivariate logistic regression analysis using variables with *p* < 0.1 was undertaken.

## 3. Results

In this study, 189 autopsies (140 males and 49 females) were enrolled, with a mean age of 61.16 years, with no statistical difference regarding the demographic data between the SMI group and the non-SMI group. Additionally, in terms of heart and coronary artery characteristics, in the SMI group there was a higher thickness of LV (*p* = 0.004), IV (*p* = 0.004), EAT LCx (*p* < 0.0001), EAT LAD (*p* < 0.0001), heart weight (*p* < 0.0001), and BMI (*p* < 0.0001) (Table 1). Moreover, there was a higher incidence of type V plaque presented in the left coronary artery (*p* = 0.01), as well as type V and type VI plaque presented in the right coronary artery (*p* = 0.02 and *p* = 0.03). Te SMI group had a higher incidence of moderate (*p* = 0.001) and severe (*p* = 0.01) valvular atherosclerosis, a higher incidence of severe left ventricle dilatation (*p* = 0.04), and a lower incidence of mild left ventricle dilatation (*p* = 0.002). In addition, at autopsy we observed a higher presence of contraction band necrosis in the SMI group (*p* < 0.0001). The rest of the variables analyzed are presented in Table 1.

**Figure 2:**
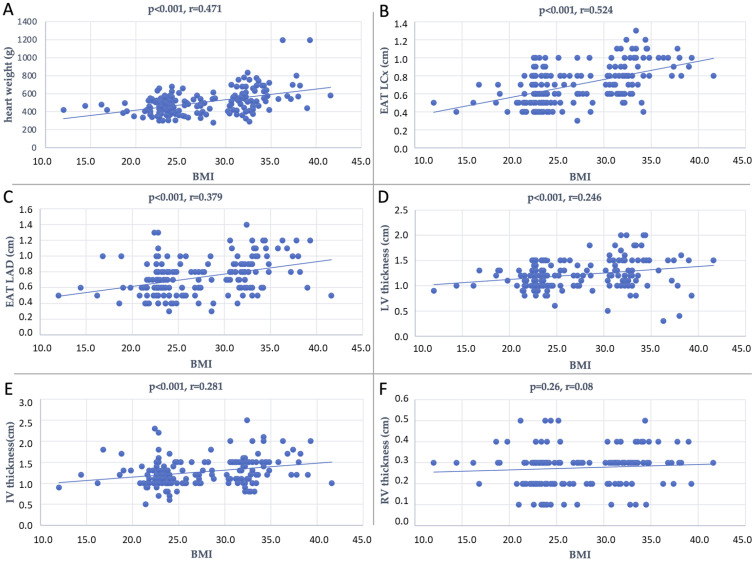
Plot representation of the dispersion of data of the correlation between BMI and heart weight (**A**), EAT LCx (**B**), EAT LAD (**C**), LV thickness (**D**), IV thickness (**E**), and RV thickness (**F**).

Figure 2 shows Spearman correlations between the BMI and heart weight (r = 0.471, *p* < 0.001), EAT LCx (r = 0.524, *p* < 0.001), EAT LAD (r = 0.379, *p* < 0.001), LV thickness (r = 0.246, *p* < 0.001), and IV thickness (r = 0.281, *p* < 0.001).

**Figure 3:**
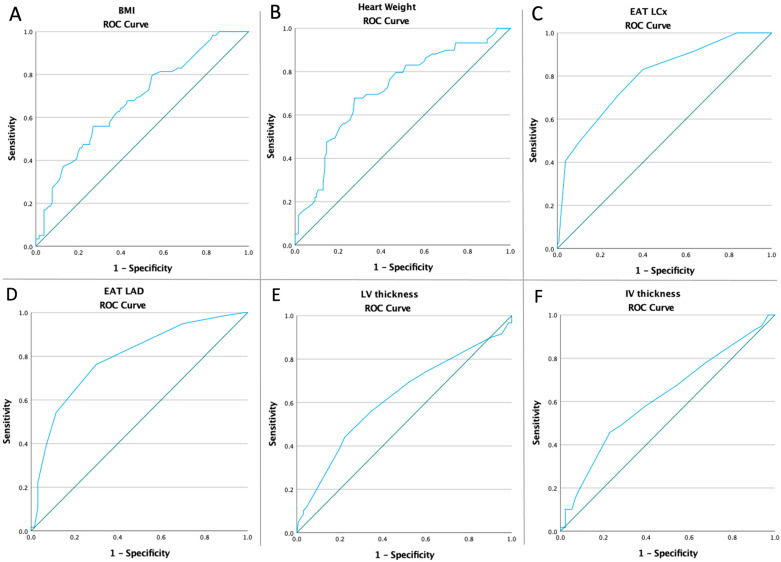
ROC curves analyzing the SMI risk in terms of BMI (**A**), and heart characteristics: (**B**) heart weight (AUC: 0.716, *p* < 0.0001), (**C**) EAT LCx (AUC: 0.796, *p* < 0.0001), (**D**) EAT LAD (AUC: 0.790, *p* < 0.0001), (**E**) LV thickness (AUC: 0.619, *p* = 0.009), and (**F**) IV thickness (AUC: 0.617, *p* = 0.01).

Receiver operating characteristic curves of all heart characteristics and BMI were computed to assess if the baseline value of all above-mentioned parameters were predictive of the risk of SMI (Figure 3). Table 2 presents the optimal cut-off value calculated using Youden’s index, the areas under the curve (AUC), sensitivity, and specificity of all parameters analyzed.

At multivariate analysis, type V plaque for the right and left coronary arteries (OR: 2.02, *p* = 0.02; and OR: 2.36, *p* = 0.01) and type VI plaque for the right coronary artery (OR: 2.75, *p* = 0.03) are independent predictors of SMI. Additionally, moderate and severe valvular atherosclerosis (OR: 3.36 and OR: 5.92, *p* = 0.002 and *p* = 0.03), as well as severe left ventricle dilatation (OR: 2.17, *p* = 0.04), strongly predict the SMI risk. In terms of heart parameters analyzed in this study, heart weight (OR: 3.64, *p* < 0.001), EAT LCx (OR: 7.35, *p* < 0.001), EAT LAD (OR: 7.50, *p* < 0.001), LV thickness (OR: 3.49, *p* = 0.01), IV thickness (OR: 3.90, *p* = 0.007), and BMI (OR: 3.07, *p* < 0.001) are all predictors of SMI risk in SCD patients. Moreover, the presence of contraction band necrosis (OR: 3.61, *p* < 0.001) is associated with a higher SMI risk, as seen in Table 3.

## 4. Discussion

SMI is a type of heart attack that occurs without any noticeable symptoms. This can make it difficult to diagnose, and SMI is more common than people may realize. Studies have shown that up to one-half of all heart attacks may be silent [18,24,25]. Also, type-1 MI, known as spontaneous MI, is characterized by symptoms such as chest pain, nausea, shortness of breath, and pain radiating to the left arm, and can occur from atherosclerosis, blood clot formation, or other types of coronary syndromes [26].

More studies have tried to report different prevalence rates according to the risk factors involved (history of cardiovascular disease, diabetic vs. non-diabetic patients). The FIELD study [27] evaluated the odds ratios of different potential risk factors for clinical MI and silent MI in diabetic patients and showed that being male, older age, having longer diabetes duration, having prior cardiovascular disease (CVD), neuropathy, higher HbA1c, albuminuria, high serum creatinine, and insulin use, all significantly predicted risk of clinical or SMI. In most studies of patients with a history of CVD, it is notable that SMI was detected by stress scintigraphy or echography or by DGE-MRI, unlike studies of patients without CVD, in which SMI was detected by routine ECG, infarction size being directly responsible for its detectability [28]. Although it is clear that ECG has its well-known limits, missing the small SMI [29], it remains the most commonly used in clinical practice when examining patients with a high risk of SMI or SCD, followed by echocardiography. Pulsed-wave Doppler tissue imaging (PWDTI) and integrated backscatter cyclic variations (IBScv) analyses have also been used to show subclinical impairment of myocardial function in young people with chronic consumption of androgenic anabolic steroids [30].

While SMI may not cause immediate symptoms, it can have serious long-term consequences, including an increased risk of SCD. The risk factors for SMI, as mentioned before, are similar to those for SCD or other types of heart disease: high blood pressure, high cholesterol, smoking, obesity, diabetes, and a family history of heart disease or SCD. While some of these risk factors are controllable through lifestyle changes and medications, others, such as family history, cannot be changed. Regarding the prognosis, studies have shown that the presence of an SMI increased the risk of mortality by a factor of 1.8–3.6 and the risk of an SCD by a factor of 1.9–3.2 [30,31,32,33,34].

Because SMI does not cause noticeable symptoms, it can be difficult to diagnose, especially in developing countries where the population lacks proper medical education regarding their healthcare and seeking prompt medical evaluation; disregards the symptoms; and blames them especially on intense physical activity. However, advances in imaging technology, such as cardiac ultrasound, CT, MRI, and proton spectroscopy methods [35,36] have long been considered gold standards in developed countries, being able to identify areas of the heart that have been damaged by a silent heart attack. However, in developing countries, the cost-effectiveness for MRI screening is likely to be unreasonable. The scarring produced after an SMI greatly increases the risk of an SCD because of its arrhythmogenic potential, with studies showing greater OR than the clinically recognized MI [17,18,19]. Unfortunately, because of the patient’s poor medical addressability, the first diagnostic is made at the autopsy table after the death is catalogued as an SCD, and we discovered limited data regarding the prevalence of SMI in individuals who died from SCD. The present findings support the view that the prevalence of SMI increases with age [16,19] and that the incidence is higher in men [37].

Because SMI can be difficult to diagnose and may not cause immediate symptoms, patients who have had a silent heart attack may not realize the severity of their condition or the importance of follow-up care. Educating patients about SMI and the importance of lifestyle changes and medications can help improve future outcomes. Healthcare professionals are also becoming more aware of SMI and its potential long-term consequences, which has led to increased efforts to identify patients who may have already had a silent heart attack and to provide appropriate follow-up care.

This study builds upon and reinforces the findings of a previous investigation, which demonstrated that higher baseline values of body mass index (OR: 4.05; *p* = 0.004), heart weight (OR: 5.47; *p* < 0.001), epicardial adipose tissue surrounding the left circumflex artery (OR: 23.72; *p* < 0.001), and epicardial adipose tissue surrounding the left anterior descending artery (OR: 21.07; *p* < 0.001) were strong independent predictors of sudden cardiac death in a case-control autopsy study comprising 80 subjects [38]. These results are also consistent with other recently published studies investigating the relationship between BMI and the risk of SCD [39].

In the future, we propose a more efficient method than the standard ECG for diagnosing SMI: after a general ECG screening, we will be able to identify individuals that are candidates for echocardiography. From this pool, we can further select individuals for an MRI scan, keeping the costs at a minimal level.

Our findings suggest that SCD is a complex condition, and its etiopathogenesis involves dynamic factors interacting with pre-existing cardiovascular pathology. The risk factors of SCD are similar to those of ischemic heart disease. By identifying and managing risk factors, as well as educating patients about the importance of follow-up care, the risk of SMI can be reduced, and outcomes can be improved, further lowering the incidence rate of SCD cases.

Although this study provides valuable insights into the potential predictors of SMI in SCD patients, there are several limitations that should be acknowledged. First, the study was conducted at a single center, which may limit the generalizability of the findings to other populations. Secondly, the study was retrospective in nature, and therefore, it was not possible to control for all potential confounding factors that may have influenced the results. Moreover, other limitations are the absence of the toxicological exams. Finally, the study focused on autopsy findings, which may not accurately reflect the clinical presentation of SMI.

## 5. Conclusions

Risk factors for SMI are similar to those for SCD or other types of heart disease. In our paper, the main results are that moderate and severe valvular atherosclerosis, severe left ventricle dilatation, heart weight, and EAT at LCx and LAD are predictors of SMI risk in SCD patients. SMI increases the risk of mortality and SCD, and its incidence is higher in men and increases with age. By identifying and managing risk factors, as well as educating patients about the importance of follow-up care, the risk of SMI can be reduced, and outcomes can be improved, further lowering the incidence rate of SCD cases.

## Figures and Tables

**Table 1:** Demographic, heart, and coronary artery characteristics of all patients.

Variables	All Patients n = 189	Non-SMI n = 130	SMI n = 59	*p* Value
Age mean ± SD	61.16 ± 10.52	60.65 ± 10.28	62.28 ± 11.06	0.33
Male/Female sex *N* (%)	140 (74.07%) 49 (25.93%)	97 (74.62%) 33 (25.38%)	43 (72.88%) 16 (27.12%%)	0.80
Heart and Coronary Artery Characteristics, mean ± SD				
BMI	27.11 ± 5.34	26.08 ± 5.13	29.38 ± 5.25	<0.0001
Heart Weight (g)	500.36 ± 134.12	469.64 ± 106.01	568.05 ± 162.89	<0.0001
EAT LCx (cm)	0.70 ± 0.20	0.63 ± 0.17	0.85 ± 0.19	<0.0001
EAT LAD (cm)	0.72 ± 0.22	0.65 ± 0.19	0.88 ± 0.20	<0.0001
LV thickness (cm)	1.21 ± 0.28	1.18 ± 0.24	1.28 ± 0.33	0.004
IV thickness (cm)	1.26 ± 0.31	1.22 ± 0.29	1.35 ± 0.34	0.004
RV thickness (cm)	0.27 ± 0.08	0.26 ± 0.07	0.28 ± 0.09	0.10
Histological Type of Left Coronary Artery Plaque, *N* (%)				
No lesion	6 (3.17%)	6 (4.62%)	-	0.21
Type I	2 (1.06%)	2 (1.54%)	-	0.58
Type II	7 (3.70%)	6 (4.62%)	1 (1.69%)	0.34
Type III	8 (4.23%)	7 (5.38%)	1 (1.69%)	0.26
Type IV	33 (17.46%)	26 (20%)	7 (11.86%)	0.17
Type V	116 (61.38%)	72 (55.38%)	44 (74.58%)	0.01
Type VI	18 (9.52%)	12 (9.23%)	6 (10.17%	0.83
Histological Type of Right Coronary Artery Plaque, *N* (%)				
No lesion	11 (5.82%)	11 (8.46%)	-	0.09
Type I	7 (3.70%)	7 (5.38%)	-	0.17
Type II	8 (4.23%)	8 (6.15%)	-	0.14
Type III	17 (8.99%)	13 (10%)	4 (6.78%)	0.48
Type IV	48 (25.40%)	35 (26.92%)	13 (22.03%)	0.20
Type V	77 (40.74%)	46 (35.38%)	31 (52.54%)	0.02
Type VI	21 (11.11%)	10 (7.69%)	11 (18.64%)	0.03
Valvular Atherosclerosis, *N* (%)				
Absent	79 (41.80%)	58 (44.62%)	21 (35.59%)	0.24
Mild	74 (39.15%)	57 (43.85%)	17 (28.81%)	0.051
Moderate	33 (17.46%)	15 (11.54%)	18 (30.51%)	0.001
Severe	7 (3.70%)	2 (1.54%)	5 (8.47%)	0.01
Left Ventricle Dilatation, *N* (%)				
Absent	16 (8.47%)	12 (9.23%)	4 (6.78%)	0.57
Mild	65 (34.39%)	54 (41.54%)	11 (18.64%)	0.002
Moderate	73 (38.62%)	45 (34.62%)	28 (47.46%)	0.09
Severe	35 (18.52%)	19 (14.62%)	16 (27.12%)	0.04
Heart autopsy findings, *N* (%)				
Contraction Band Necrosis	47 (24.87%)	22 (16.92%)	25 (42.37%)	<0.0001
Hypoplastic Coronary Artery Disease	27 (14.29%)	16 (12.31%)	11 (18.64%)	0.25
Cardiac Lipomatosis	33 (17.46%)	23 (17.69%)	10 (16.95%)	0.90
Fibrinous Pericarditis	17 (8.99%)	12 (9.23%)	5 (8.47%)	0.86
Coronary Bridging	16 (8.47%)	9 (6.92%)	7 (11.86%)	0.26

**Table 2:** Optimal cut-off values and characteristics of all parameters analyzed with ROC curves.

Variables	Cut-Off	AUC	Std. Error	95% CI	Sensitivity	Specificity	*p* Value
Silent Myocardial Infarction							
BMI	30.5	0.677	0.042	0.595–0.758	55.9%	73.1%	<0.0001
Heart Weight	502.5	0.716	0.041	0.636–0.795	69.5%	66.9%	<0.0001
EAT LCx	0.65	0.796	0.035	0.728–0.865	83.1%	60%	<0.0001
EAT LAD	0.75	0.790	0.036	0.720–0.861	76.3%	70%	<0.0001
LV thickness	1.25	0.619	0.046	0.528–0.709	55.9%	65.4%	0.009
IV thickness	1.25	0.617	0.045	0.528–0.706	57.6%	60.8%	0.01

**Table 3:** Multivariate analysis for all parameters regarding the SMI risk.

	Silent Myocardial Infarction		
	OR	95% CI	*p* value
	Left Coronary Artery		
Type V plaque	2.36	1.19–4.66	0.01
	Right Coronary Artery		
Type V plaque	2.02	1.08–3.77	0.02
Type VI plaque	2.75	1.09–6.89	0.03
	Valvular Atherosclerosis		
Mild	0.51	0.26–1.004	0.052
Moderate	3.36	1.55–7.28	0.002
Severe	5.92	1.11–13.49	0.03
	Left Ventricle Dilatation		
Mild	0.32	0.15–0.67	0.003
Moderate	1.70	0.91–3.19	0.09
Severe	2.17	1.02–4.61	0.04
Contraction Band Necrosis	3.61	1.81–7.20	<0.001
BMI	3.07	1.62–5.81	<0.001
Heart Weight	3.64	1.88–7.03	<0.001
EAT LCx	7.35	3.41–15.8	<0.001
EAT LAD	7.50	3.69–15.21	<0.001
LV thickness	3.49	1.26–12.29	0.01
IV thickness	3.90	1.44–10.56	0.007
RV thickness	1.66	0.40–6.92	0.13

## Data Availability

Not applicable.
